# Effect of Non-depolarizing Muscle Relaxants Rocuronium Versus Vecuronium in the Assessment of Post-Succinylcholine Complications in Surgeries Under General Anesthesia: A Randomized Double-Blind Study at a Tertiary Care Hospital

**DOI:** 10.7759/cureus.19793

**Published:** 2021-11-21

**Authors:** Laxman K Senapati, Krishna P Battini, Pulak P Padhi, Priyadarsini Samanta

**Affiliations:** 1 Department of Anesthesia, Kalinga Institute of Medical Sciences, KIIT Deemed to be University, Bhubaneswar, IND; 2 Department of Physiology, Kalinga Institute of Medical Sciences, KIIT Deemed to be University, Bhubaneswar, IND

**Keywords:** myalgia, fasciculations, intubation, succinylcholine, vecuronium, rocuronium, nondepolarizing muscle relaxants

## Abstract

Background and objective

Several drugs have been used to prevent or attenuate succinylcholine-induced muscle fasciculations and myalgia. We designed the present study to evaluate the efficacy of rocuronium (ROC) and vecuronium (VEC) in preventing succinylcholine-induced fasciculations and postoperative myalgia (POM) in patients undergoing surgery under general anesthesia.

Materials and methods

After obtaining written informed consent, 125 patients were randomly selected to receive either ROC 0.06 mg/kg or VEC 0.01 mg/kg, with both diluted up to 2 ml, 90 seconds before the administration of propofol followed by succinylcholine. A standardized balanced anesthetic technique was used for all patients. The intensity of fasciculations and intubating conditions were assessed using a 4-point rating scale. All patients were evaluated up to the third postoperative day for the presence of POM, the severity of which was graded on a 4-point scale.

Results

The incidence of post-succinylcholine fasciculations during induction was nil in 74.58% of patients in the ROC group and 51.52% in the VEC group. Mild fasciculation was seen in 22.03% in the ROC group and 33.33% in the VEC group. Moderate fasciculation was seen in 3.39% and 15.15% in ROC and VEC groups respectively. When comparing both the groups, a significant decrease (p=0.015) in intraoperative fasciculation was observed in the ROC group than in the VEC group. Both drugs provided good intubating conditions without any statistical significance and with an overall intubating score of 8-9 in both groups as per Lund. On day one, 91.53% (n=54) of the ROC group and 65.15% (n=43) of the VEC group patients did not have any myalgia symptoms. Mild myalgia was observed in 8.47% (n=5) in the ROC group and 31.82% (n=21) in the VEC group, and only 1.8% had moderate myalgia in the VEC group. The results of the study showed that POM was significantly decreased in the ROC group than in the VEC group on day one (p=0.001). The incidence of POM on day two was significantly low in both groups. There was no statistical significance between the two groups based on Fisher's exact test (p=1.000). None of the patients had myalgia on day three.

Conclusion

Our results showed that the incidence and severity of fasciculations and POM were significantly decreased by pretreatment with ROC in contrast to that with VEC. Hence, ROC is a better option than VEC to combat succinylcholine-related complications like fasciculation and myalgia.

## Introduction

During rapid sequence intubation, succinylcholine plays a pivotal role in administering general anesthesia as it produces profound and quick neuromuscular block within 30 to 60 seconds, which lasts for three to five minutes [1]. It is a life-saving drug in cases of difficult intubation or failed intubation scenarios owing to its short half-life.

However, its helpfulness is often accompanied by the persistent manifestation of postoperative myalgia (POM) besides other plethora of side effects such as persistent neuromuscular blocks in patients with pseudocholinesterase deficiency, malignant hyperthermia, and rhabdomyolysis in patients with myopathy [2]. The incidence of succinylcholine-induced myalgia ranges from 20 to 80% [3]. It usually lasts from two or three days to as long as a week and is most notable in the muscles of the neck, back, and abdomen [4,5]. In the contemporary practice of anesthesia, myalgia is insufferable even though it perishes on its own [6].

To decrease the incidence and severity of myalgia, various treatment methods have been recommended, which includes lignocaine [7], diazepam, ketorolac, diclofenac [8], gabapentin, remifentanil, cisatracurium [9], d-tubocurarine, pancuronium, vecuronium (VEC) [10], rocuronium (ROC) [11], and atracurium [12]. Among these options, pretreatment with a small dose of the non-depolarising neuromuscular drug before succinylcholine administration is the most effective [13]. The efficacy of the pretreatment is determined by the choice of the non-depolarizing agent [14,15], the extent of prejunctional receptor block, the time gap between the administration of the pretreatment agent and succinylcholine, and the speed of onset of the non-depolarising drug.

As opposed to all the commonly used non-depolarising agents, the onset of action of ROC is very rapid and it generates perfect intubating condition within 60 seconds [16]. The frequency and severity of fasciculations are markedly reduced by pretreatment with ROC 90 seconds before succinylcholine administration [17]. Schreiber et al. [18] have established that ROC and non-steroidal anti-inflammatory agents are the prime agents to prevent fasciculation and myalgia induced by succinylcholine, based on a meta-analysis of randomized trials.

To date, there have been limited studies on the occurrence and severity of myalgia up to the third postoperative day following succinylcholine administration. Hence, the present research was undertaken to compare the effect of pretreatment with ROC, the near-ideal muscle relaxant, with that with VEC, an already established neuromuscular blocking agent for post-succinylcholine complications such as fasciculations and myalgia up to the third postoperative day. We also tried to assess if there were any differences between the intubating conditions produced by the two drugs.

## Materials and methods

Study participants and study design

Considering an effect size of 0.5, power (1-β) of 0.80, a 5% level of significance, and an attrition rate of 5%, the calculated total sample size was 132, i.e., 66 in each group. After obtaining approval from the Institutional ethics committee (KIIT/KIMS/IEC/128/2019, CTRI no: CTRI/2019/10/021820) and receiving written informed consent, a randomized double-blind study was conducted with 125 patients (excluding seven patients lost to follow-up) from September 2019 to September 2021; the patients were categorized into in two groups (59 in the ROC group and 66 in the VEC group). The study was conducted at the tertiary care center of Pradyumna Bal Memorial Hospital, Kalinga Institute of Medical Sciences (KIMS), Bhubaneswar, Odisha. Patients aged 16-80 years, with the American Society of Anesthesiologists (ASA) physical status I and II, who were undergoing elective surgery under general anesthesia with endotracheal intubation of a duration of at least 30 minutes were included in our study. Patients having body mass index (BMI) ≥30, history of allergy to any particular drugs used in the study, pregnant patients, those with post-burn, post-trauma, and other hyperkalemic states, those with an anticipated difficult airway, those with hepatic or renal dysfunction and increased intraocular or intracranial pressure were excluded.

Randomization was done by a computer-generated randomization list and allocation was done by sequentially numbered opaque envelopes to one of the two groups (ROC group and VEC group). The envelope was opened by an anesthetist just before administering the drug. A double-blinding technique was followed, where an anesthesiologist who was not part of the study prepared the solution in a standardized volume of 2-ml syringes. The observer and the patients were unaware of the pretreatment used.

Anesthetic technique

Injection glycopyrrolate 0.2 mg IV, injection midazolam 1 mg IV, and injection nalbuphine 0.1 mg/kg IV were administered as premedication; the standard anesthetic technique was followed in all patients, and electrocardiography, oxygen saturation, and non-invasive blood pressure monitoring were carried out. The two groups were as follows: the ROC group received 0.06 mg/kg of ROC IV diluted up to 2 ml and the VEC group received 0.01 mg/kg of VEC IV diluted up to 2 ml. After 90 seconds, the patients were induced with propofol 1.5-2 mg/kg IV and succinylcholine 1.5 mg/kg IV. Direct laryngoscopy was performed one minute after the administration of succinylcholine and the patient's trachea was intubated via oral route in the sniffing position with an endotracheal tube of appropriate size. The laryngoscopy was carried out by an experienced anesthesiologist in all patients to assess the intubating conditions; the anesthesiologist was blinded to the pretreatment group.

Intraoperatively, anesthesia was maintained with isoflurane (0.5-1.5% end-tidal concentrations) along with nitrous oxide 60% in oxygen and intermittent doses of VEC or ROC. Patients were ventilated in the volume control mode of ventilation to achieve an end-tidal CO_2_ concentration of 35-40 mmHg. Intraoperative analgesia was provided with IV paracetamol 1 gm. At the end of the surgery, residual neuromuscular blockade was reversed with injection glycopyrrolate 10 mcg/kg and injection neostigmine 0.05 mg/kg. After complete recovery, all patients were extubated and shifted to the post-anesthesia care unit (PACU).

Assessment of fasciculations, myalgia, and intubating conditions

The severity of fasciculation was assessed on a 4-point scale (Foster, 1960) [19], by an anesthesiologist blinded to the patient's group assignment, where 0=nil fasciculations; 1=mild, fine fasciculations of the eyes, neck, face, or fingers without limb movement; 2=moderate fasciculations occurring at more than two sites or obvious limb movement; and 3=vigorous or severe, sustained, and widespread fasciculations in the trunk and limbs.

Intubating con­ditions were assessed as per guidelines by Lund (1970) [20], where a score of 3=good jaw relaxation, vocal cords open, and immobile with no response to intubation; a score of 2=moderate jaw relaxation, vocal cords moving and slight diaphragmatic movements in response to intubation; a score of 1=minimum jaw relaxation, vocal cords closing, and mild coughing in response to intubation; and a score of 0=poor jaw relaxation, closed vocal cords, and severe coughing in response to intubation. The total score for intubation was categorized as follows: excellent=8-9, good=6-7, fair=3-5, and poor=0-2 (Lund, 1970) [20].

Myalgia was also graded on a 4-point scale (White, 1962) [21] on day one, day two, and day three postoperatively and was assessed by another investigator who was unaware of the group details, where nil=no muscle pain; mild=muscle stiffness or pain, when specifically enquired in the nape of the neck, or the shoulders and lower chest on deep breathing; moderate=muscle stiffness and pain spontaneously complained of by the patient that needs analgesics; and severe=incapacitating generalized muscle stiffness or pain.

Statistical analysis

IBM SPSS Statistics software version 23.0 (IBM, Armonk, NY) was used for all analyses. All categorical variables were expressed as frequency and percentages. The continuous variables of the data were presented as mean ±standard deviation. The Fisher’s exact test was applied to compare and assess the significance of the difference in the mean values of variables between the two groups (the ROC group and the VEC group). A p-value of <0.05 was considered statistically significant.

## Results

**Figure 1:**
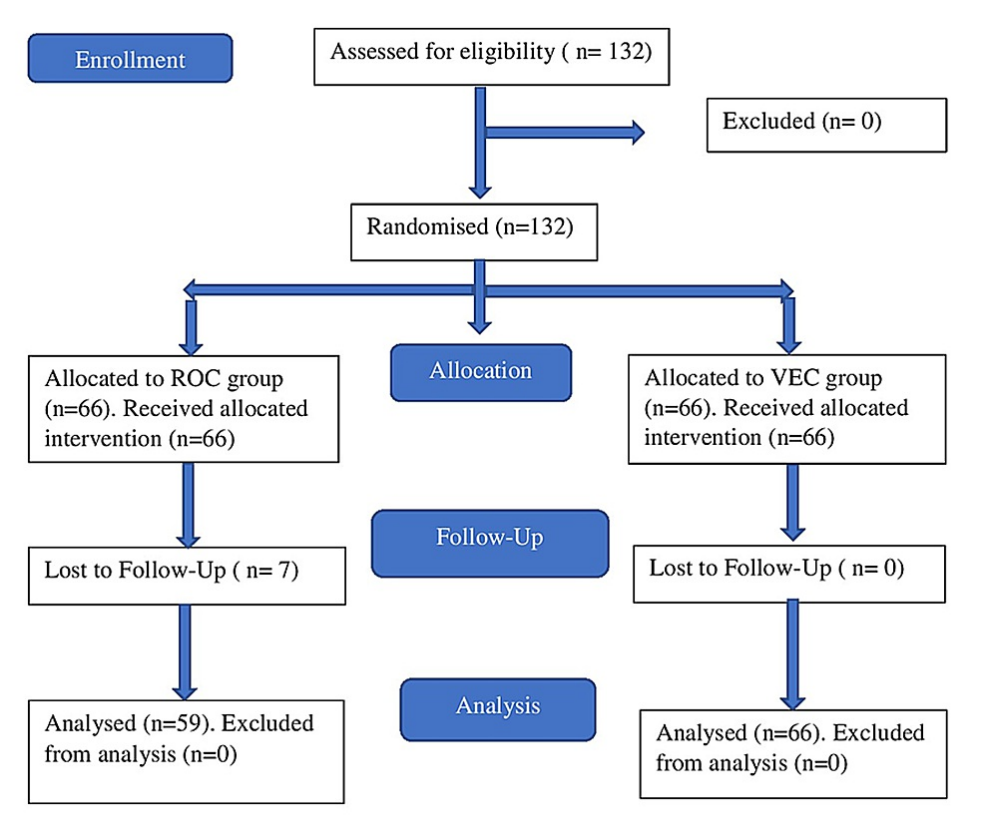
CONSORT study flow chart CONSORT: Consolidated Standards of Reporting Trials; ROC: rocuronium group; VEC: vecuronium group

A total of 132 patients were eligible for the study, but 125 patients were finally included in the study since seven patients in the ROC group were lost to follow-up (Figure 1).

Demographic variables

A total of 125 patients were included in the study to observe the incidence of fasciculation and myalgia. No significant differences in terms of age, weight, ASA status, MP grade, and dose of succinylcholine were observed between the two groups (Tables 1-5).

**Table 1:** Age and weight distribution between two groups ROC: rocuronium group; VEC: vecuronium group; SD: standard deviation

	ROC (n=59)	VEC (n=66)	P-value
Age, years, mean ±SD	39.45 ± 11.85	39.59 ±11.78	0.949
Weight, kg, mean ±SD	61.94 ±10.96	63.4 ±11.22	0.464

**Table 2:** Gender distribution between two groups ROC: rocuronium group; VEC: vecuronium group

Gender	Group		Total, n (%)	P-value
	ROC, n (%)	VEC, n (%)		
Male	22 (37.3)	33 (50)	55 (44)	0.153
Female	37 (62.7)	33 (50)	70 (56)	
Total	59	66	130	

**Table 3:** ASA grade distribution between two groups ASA: American Society of Anesthesiologists; ROC: rocuronium group; VEC: vecuronium group

ASA grade	Group		Total, n (%)	P-value
	ROC, n (%)	VEC, n (%)		
I	39 (66.1)	41 (62.1)	80 (64)	0.643
II	20 (33.9)	25 (37.9)	45 (36)	
Total	59	66	125	

**Table 4:** MP grade distribution between two groups ROC: rocuronium group; VEC: vecuronium group

MP grade	Group		Total, n (%)	P-value
	ROC, n (%)	VEC, n (%)		
1	33 (55.9)	30 (45.5)	63 (50.4)	0.242
2	26 (44.1)	36 (54.5)	62 (49.6)	
Total	59	66	125	

**Table 5:** Dose of succinylcholine between two groups ROC: rocuronium group; VEC: vecuronium group; SD: standard deviation

	ROC	VEC	P-value
Dose of succinylcholine, mg, mean ±SD	92.31 ±16.15	95.72 ±15.01	0.223

Incidence of fasciculations

Out of 125 patients, 62.4% (n=78) had no fasciculations while 28% (n=35) had grade 1 fasciculations and 9.6% (n=12) had grade 2 fasciculations. Grade 1 and grade 2 fasciculations were considered to be mild and moderate respectively (Table 6).

**Table 6:** Gradation of intraoperative fasciculation based on incidences of fasciculation

Grades of intraoperative fasciculation	Number of patients	Percentage
Nil	78	62.40
Mild	35	28.00
Moderate	12	9.60
Severe	0	0

Comparative Incidence of Fasciculations in ROC vs. VEC

The incidence of intraoperative fasciculations was nil in 74.58% of patients in the ROC group and 51.52% in the VEC group. Mild fasciculation was seen in 22.03% in the ROC group and 33.33% in the VEC group. Moderate fasciculation was seen in 3.39% and 15.15% in ROC and VEC groups respectively. When comparing both the groups, a significant decrease (p=0.015) in intraoperative fasciculation was observed in the ROC group than in the VEC group (Table 7).

**Table 7:** Percentage of the severity of fasciculations in each group ROC: rocuronium group; VEC: vecuronium group

Grades of intraoperative fasciculation	ROC (%)	VEC (%)	Total (%)
Nil	74.58	51.52	62.40
Mild	22.03	33.33	28.00
Moderate	3.39	15.15	9.60

Intubation condition

An intubation score of 9 as per Lund [20] was observed in 55% of the ROC group and 50% of the VEC group of patients. There was no statistically significant difference with regard to the mean intubation score between the two groups. We were able to intubate most of the patients with ease since both the drugs led to excellent intubating conditions (Tables 8, 9).

**Table 8:** Intubation condition scores between two groups ROC: rocuronium group; VEC: vecuronium group; SD: standard deviation

Intubation score	ROC		VEC	
	Number of patients	Percentage	Number of patients	Percentage
10	_	_	_	_
9	32	55%	33	50%
8	16	27%	18	28%
7	8	13%	7	11%
6	3	5%	6	9%
5	_	_	2	2%
Less than 4	_	_	_	_
Total		59		66
Mean ±SD	8.30 ±0.89			8.12 ±1.11

**Table 9:** Intubating condition ROC: rocuronium group; VEC: vecuronium group

Intubating condition	ROC		VEC	
	Number of patients	Percentage	Number of patients	Percentage
Excellent	47	80%	50	76%
Good	12	20%	15	22%
Fair	_	_	1	2%
Poor	_	_		
Total	59		66	

Incidence of myalgia

The incidence of myalgia was monitored on three consecutive days (days one, two, and three) postoperatively. Among 125 patients, the incidence of myalgia was nil in 77.6% of patients on day one, and on the second day, 99.2% had no incidence of myalgia. Mild myalgia was observed in 20.8% on day one, and only 0.8% had mild myalgia on the second postoperative day (Table 10). The incidence of moderate myalgia was 1.6% and 0.8% on days one and two respectively. None of the patients in the sample group had severe myalgia. The incidence of myalgia was zero in both groups on day three.

**Table 10:** Incidence of postoperative myalgia with mild and moderate grades

Day 1	Frequency	Percentage	Cumulative percentage
Nil	97	77.60	77.60
Mild	26	20.80	98.40
Moderate	2	1.60	100.00
Day 2	Frequency	Percentage	Cumulative percentage
Nil	124	99.20	99.20
Mild	1	0.80	100.00

Comparative Incidence of Myalgia in ROC vs. VEC Group of Patients on Day One

The incidence of POM was recorded for up to three days between two groups (ROC and VEC). On day one, 91.53% (n=54) of the ROC group and 65.15% (n=43) of the VEC group of patients did not have any myalgia symptoms. Mild myalgia was observed in 8.47% (n=5) in the ROC group and 31.82% (n=21) in the VEC group, and only 1.8% had moderate myalgia in the VEC group. The results of the study showed that POM was significantly decreased in the ROC group than in the VEC group (p=0.001) (Table 11).

**Table 11:** Comparative incidences of postoperative myalgia on day one ROC: rocuronium group; VEC: vecuronium group

Grades of myalgia	ROC (%)	VEC (%)	Total (%)
Nil	91.53	65.15	77.60
Mild	8.47	31.82	20.80
Moderate	0.00	3.03	1.60

Comparative Incidence of Myalgia in ROC vs. VEC Group of Patients on Day Two

The incidence of POM on day two was significantly less in both groups. There was no statistically significant difference between the two groups based on Fischer's exact test (p=1.000) (Table 12).

**Table 12:** Comparative incidences of postoperative myalgia on day two ROC: rocuronium group; VEC: vecuronium group

Grades of myalgia	ROC (%)	VEC (%)	Total (%)
Nil	100.00	98.48	99.20
Mild	0.00	1.52	0.80

## Discussion

VEC was described as the best pretreatment agent in preventing succinylcholine-induced complications [14,15]; in light of this, we engaged in a study to compare ROC with VEC. We used a VEC dose of 1 mg based on previous observations [15] and the dose of ROC was determined to be 6 mg based on potency ratio. The administration of pretreatment agents both on a fixed drug regimen [15,22] and on the basis of weight [23] has been postulated and we preferred dosing as per weight to maintain uniformity. Kim et al. [11] in their study used different precurarization doses of ROC, of which a dose of 0.06 mg/kg resulted in less incidence of fasciculations, with the acceptable onset of actions. Hence, we took the precurarization dose of ROC to be 0.06 mg/kg as also proposed by Fukano et al. [24], Subramaniam et al. [25], and Pinchak et al. [26].

Of late, there has been a lot of debate regarding the time interval between the administration of pretreatment agents and succinylcholine. Intervals of two, three, and four minutes or longer have been proposed [21,27,28]. But these prolonged intervals are not only impractical with busy operating room lists but may also expose the awake patient to the potentially unpleasant experiences of difficulty in swallowing, breathing, and muscle weakness, and may also lead to desaturation of the patient because of the longer apnea time. To avoid these hazards, we chose 90 seconds of rapid precurarizing time, which reduces the possibility of exposing the patients to the side effects of precurarization.

Fasciculations

The severity of fasciculation was comparatively low in the ROC group than in the VEC group. In the ROC group, incidence of grade 1 (mild) fasciculation was 10.4% (n=13) and that of grade 2 (moderate) was 1.6% (n=2). The incidence of grade 1 and grade 2 fasciculations in the VEC group was 17.6% (n=22) and 8% (n=10) respectively. It was discovered that the efficacy of preventing succinylcholine-induced fasciculations relies upon the level of affinity of non-depolarising muscle relaxants for prejunctional choline receptors [29]. Higher affinity was found with ROC, which explains its better effectiveness.

In a study conducted by Joshi et al. [30], the incidence of fasciculations was 24% with ROC and 48% with VEC, with a 100% incidence rate in the control group (normal saline). In a study by Abbas et al. [31] using 0.1 mg/kg of ROC as pretreatment, 100% (mild to severe) fasciculations were noticed in the saline group as compared to 13.3% (mild) in the ROC group. Abraham et al. [32] found that fasciculations after succinylcholine administration was less in the ROC group (0.06 mg/kg) compared to the VEC group (0.04 mg/kg), which also correlates with our study.

Demers-Pelletier et al. [33] reported in their study that post-succinylcholine fasciculations were more intense in saline groups as compared to pretreatment with ROC (p<0.001) group. Findlay and Spittal [34] found that fasciculation in the group precurarized with VEC was more when compared to ROC (p<0.01). In a study by Martin et al. [35], it was seen that ROC was the best option to prevent muscular fasciculations following succinylcholine injection among d-tubocurarine, VEC, mivacurium, atracurium, and ROC. Joshi et al. [9] ascertained that fasciculations were observed less frequently (p<0.05) in the d-tubocurarine and ROC groups compared with the placebo and cisatracurium groups. Our findings are in line with all of the above studies.

Intubating conditions

In our study, it was observed that pretreatment with either ROC or VEC provided good intubating conditions without any statistically significant difference, with an overall intubation score of 8.30 ±0.89 in the ROC group and 8.12 ±1.11 in the VEC group.

O'Sullivan et al. [36], Findlay and Spittal [34], Tsui et al. [37], and Martin et al. [35] utilized ROC and VEC pretreatment before the administration of succinylcholine for endotracheal intubation. They could not find any dissimilarity in intubation conditions in their patients, which endorses the findings of our study.

Postoperative myalgia

In our study, the overall occurrence of myalgia on postoperative day one was found to be 21.8% and negligible on day two and day three. The severity of POM was less with ROC than VEC. We observed that the incidence of mild myalgia in the ROC group was 4% on day one, and it was 16% in the VEC group, which was statistically significant (p=0.001). The incidence of moderate myalgia in the VEC group was 1.6% on day one and zero in the ROC group, which was also statistically significant (p=0.001). The myalgia on postoperative day two and day three did not show any statistical significance between the two groups.

In a study conducted by Joshi et al. [30], the incidence of mild to moderate myalgia was found to be higher in the VEC group when compared to the ROC group. Our findings concur with those of O'Sullivan et al. [36] and Erkola [14]. Findlay and Spittal [34] could not find any statistically significant difference between the ROC and VEC groups on the occurrence of myalgia on the third postoperative day, which concurs with our study.

Waters and Mapleson [38] put forward that myalgia occurs because of the damage produced in muscles by the unsynchronized contraction of adjacent muscle fibers just before the onset of paralysis leading to shearing of connective tissues, the other reasons being the release of prostaglandins and electrolyte imbalance. But the association between fasciculations and myalgia has not been elucidated by many researchers. Hence, pretreatment with non-depolarizing agents subdues the adverse action of succinylcholine at the neuromuscular junction.

Limitations

The limitation of our study is that we could have included a saline group as controls and compared both the drugs with it. But we felt that it would be unethical to use saline instead of proven drugs in patients and thereby put them in jeopardy of the potential complications of succinylcholine.

## Conclusions

The occurrence and severity of fasciculations were significantly less in the ROC group in contrast with the VEC group. Both of the drugs produced excellent intubating conditions. Similarly, the incidence and seriousness of POM on day one were remarkably less in the ROC group than in the VEC group. But on the second postoperative day, the incidence of myalgia was similar in both groups. No patients complained of myalgia on the third postoperative day. Hence, ROC is better than VEC to combat succinylcholine-induced complications like fasciculation and myalgia and it produced rapid precurarization in one minute, thereby minimizing the unpleasant experience of partial neuromuscular blockade from precurarization. Reduced myalgia promotes early patient ambulation, leads to fewer postoperative complications like deep vein thrombosis, and results in faster discharge from the hospital. We propose that ROC is cost-effective due to reduced postoperative analgesic requirements resulting in early ambulation. Even though it is not standard practice, the use of ROC is advocated before succinylcholine to negate the side effects while taking advantage of its plethora of benefits in various elective surgeries.

Even with the advent of newer drugs with better pharmacological profiles, the advantages of succinylcholine cannot be underestimated. The rapid and excellent intubating conditions achieved by succinylcholine are unparalleled. Hence, this study could also be extrapolated to patients needing succinylcholine for rapid sequence induction in conditions like GERD and patients with a full stomach and anticipated difficult airways.

## Appendices

Author Contribution: Study Design: Laxman K. Senapati, Pulak P. Padhi. Study Registration: Krishna P. Battini. Patient Recruitment and Data Collection: Laxman K. Senapati, Krishna P. Battini. Clinical Operation: Laxman K. Senapati, Pulak P. Padhi. Manuscript Writing and Statistical Analysis: Priyadarsini Samanta, Laxman K. Senapati.
